# Adversarial Attacks on Intrusion Detection Systems in In-Vehicle Networks of Connected and Autonomous Vehicles

**DOI:** 10.3390/s24123848

**Published:** 2024-06-14

**Authors:** Fatimah Aloraini, Amir Javed, Omer Rana

**Affiliations:** 1 School of Computer Science and Informatics, Cardiff University, Cardiff CF10 3AT, UK; javeda7@cardiff.ac.uk (A.J.); ranaof@cardiff.ac.uk (O.R.); 2 College of Sciences and Humanitie, Shaqra University, Shaqra 11911, Saudi Arabia

**Keywords:** adversarial machine learning, connected and autonomous vehicle, controller area network, in-vehicle network, cybersecurity

## Abstract

Rapid advancements in connected and autonomous vehicles (CAVs) are fueled by breakthroughs in machine learning, yet they encounter significant risks from adversarial attacks. This study explores the vulnerabilities of machine learning-based intrusion detection systems (IDSs) within in-vehicle networks (IVNs) to adversarial attacks, shifting focus from the common research on manipulating CAV perception models. Considering the relatively simple nature of IVN data, we assess the susceptibility of IVN-based IDSs to manipulation—a crucial examination, as adversarial attacks typically exploit complexity. We propose an adversarial attack method using a substitute IDS trained with data from the onboard diagnostic port. In conducting these attacks under black-box conditions while adhering to realistic IVN traffic constraints, our method seeks to deceive the IDS into misclassifying both normal-to-malicious and malicious-to-normal cases. Evaluations on two IDS models—a baseline IDS and a state-of-the-art model, MTH-IDS—demonstrated substantial vulnerability, decreasing the F1 scores from 95% to 38% and from 97% to 79%, respectively. Notably, inducing false alarms proved particularly effective as an adversarial strategy, undermining user trust in the defense mechanism. Despite the simplicity of IVN-based IDSs, our findings reveal critical vulnerabilities that could threaten vehicle safety and necessitate careful consideration in the development of IVN-based IDSs and in formulating responses to the IDSs’ alarms.

## 1. Introduction

As we move toward the future, connected and autonomous vehicles (CAVs) are expected to form the backbone of transportation systems. The global market for connected vehicles is expected to reach approximately USD 121 billion by 2025 [1]. Leading car manufacturers such as General Motors, Toyota, Volvo, Volkswagen, and BMW, as well as tech-centric companies such as Google, Uber, and Tesla, are at the forefront of the development of autonomous vehicles [2]. This explosive progress is driven by breakthroughs in machine learning (ML), especially deep learning (DL), which are crucial in creating sophisticated autonomous and connected vehicular ecosystems. The primary role of ML in CAVs involves enhancing decision-making capabilities, both in terms of environmental perception and in strengthening defenses against cybersecurity threats.

However, integrating ML/DL into CAVs introduces substantial cybersecurity concerns. Studies, including [3,4], have exposed the vulnerabilities of ML/DL models to a unique category of threats known as ‘adversarial attacks’. These are techniques that manipulate input data, such as altering road signs in imperceptible ways, causing ML models to misclassify and respond inappropriately. Such attacks are especially critical in CAV contexts, where they could lead to life-threatening situations, loss of trust in technology, financial damage, and potential legal liabilities for manufacturers.

In light of this, numerous studies [5,6,7,8,9,10,11,12,13,14] have addressed the susceptibility of ML/DL models in CAVs to adversarial manipulation, particularly focusing on perception models like road sign classification and object detection models. Mbow et al. [15] and Limbasiya et al. [16] have highlighted the critical need to investigate the susceptibility of ML models in CAVs to adversarial attacks, identifying this as a significant research gap. Rajapaksha et al. [17] further emphasized the need to investigate the robustness of intrusion detection systems (IDSs) within in-vehicle networks (IVNs) against such attacks. Yet, to the best of our knowledge, no study has extensively explored the implications of adversarial attacks on models that govern cybersecurity functions within vehicles.

Building on this foundation, this study aimed to explore adversarial attacks against IDS models deployed within CAVs. Our primary motivation stems from the fact that cybersecurity models, especially IDSs, are deployed in critical locations, that is, IVNs, which are internal networks that connect various components of vehicles and serve as the backbone of these vehicles. If adversaries successfully compromise these IDSs, they could gain control over the vehicle without having to manipulate its environmental surroundings. Furthermore, the controller area network (CAN) protocol, the main communication protocol used in IVNs, originally designed for isolated vehicles, lacks essential security measures. This makes IDSs in an IVN more accessible compared to those in a traditional or Internet of Things (IoT) network setting, increasing the potential for adversarial attacks. Previous research has shown the feasibility of remotely accessing an IVN in several modern vehicles such as the Jeep Cherokee [18], Tesla [19], and BMW [20], particularly as these vehicles evolve toward a higher degree of connectivity and autonomy, such as having internet connections. Lastly, in recent developments, considerable emphasis has been placed on enhancing the accuracy of IDSs for IVNs. However, the primary focus on accuracy may offer a false sense of security. As highlighted by a recent survey [17], the prevalent literature largely neglects the robustness of these systems against adversarial cyber attacks. This oversight suggests that while IDS solutions might demonstrate high detection rates, they could ironically become the weakest link in IVNs if their susceptibility to adversarial attacks is not addressed. Consequently, deploying IDSs without this crucial consideration not only fails to protect the vehicle but also potentially escalates the risk of vehicle manipulation.

As a result, we proactively address the susceptibility of these IDS models to adversarial attacks. To our knowledge, this is the first study to explore how adversarial manipulation affects cybersecurity models, especially IDS models, within CAVs. It presents several key contributions:

- The introduction of a novel method for generating adversarial examples to assess IDSs in IVNs.
- The provision of insights into the potential success of adversarial attacks in real-world IVN scenarios through behavioral analysis.
- The proposal of a comprehensive threat model specifically tailored for IDSs in IVNs, focusing on adversarial methods.
- The identification of effective adversarial techniques that generate examples capable of compromising IDSs in IVNs, thereby posing significant safety and security risks.
- The examination of the impact of manipulated benign IVN network frames, altered to appear malicious, on the performance of a targeted IDS, highlighting the risk of false alarms triggering unintended defensive mechanisms.

We evaluated the proposed adversarial attack on two IVN-based IDS models: A baseline-IDS and a state-of-the-art IDS, MTH-IDS [21]. Our method significantly reduced the F1 score of the baseline-IDS from 95% to 38% and that of the state-of-the-art IDS from 97% to 79%. By demonstrating a systematic approach to attacking ML-based IDSs, we establish a foundation for assessing the robustness of future IDS solutions and for enhancing the response mechanisms of IVN systems.

## 2. Background

This section outlines the preliminary knowledge of the main concepts used in this paper.

### 2.1. In-Vehicle Network

The IVN, an essential component of modern vehicles, is a system of interconnected components within a vehicle that facilitates communication and data exchange [2,16]. The IVN typically consists of electronic control units (ECUs). ECUs are small standalone devices that are used to control specific functions within a vehicle, such as engine control, transmission, safety, and many others [22]. Each ECU manages a specific set of functions and operates independently of other ECUs. However, they are all connected to the IVN and can communicate with each other to coordinate their actions. For example, a seemingly straightforward braking process requires interaction between the engine control, the anti-lock brake system, and the instrument panel cluster.

In addition, the IVN is connected to the on-board diagnostic port, known as OBD-II. In this context, ‘II’ indicates the second version of the onboard diagnostic port, which is a standardized interface allowing access to the vehicle diagnostic system and real-time IVN data.

**Figure 1:**
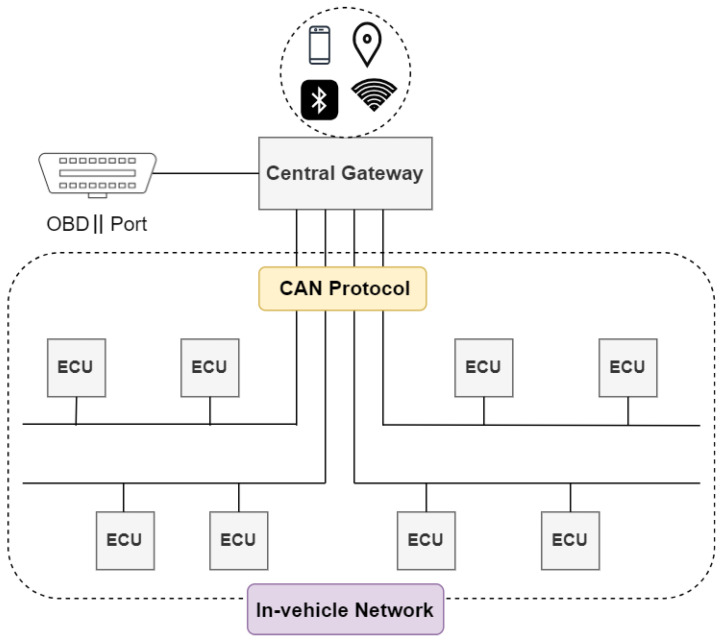
Overview of in-vehicle network architecture in connected and autonomous vehicles.

In the context of connected and autonomous vehicles, as depicted in Figure 1, connectivity is expanded. In addition to the IVN that connects different types of ECUs to exchange operational messages, these vehicles also establish connections with the outside world using technologies such as cellular interfaces to offer improved services [16].

### 2.2. Controller Area Network Protocol

The CAN protocol, also known as CAN-Bus, is described as ‘a serial communication protocol that efficiently supports distributed real-time control with a very high level of security’ [23]. The CAN operates by broadcasting its frames, allowing all nodes connected to the CAN bus to receive the transmitted frames [24]. The CAN protocol has several key features, such as an effective data rate and cost, excellent error detection, and a bus topology that results in high-integrity real-time communications for the IVN [22,23,25]. Due to its advantageous features, the CAN is widely used in the automotive industries as the primary protocol for IVNs to transmit data between various nodes [22,23,25].

**Figure 2:**
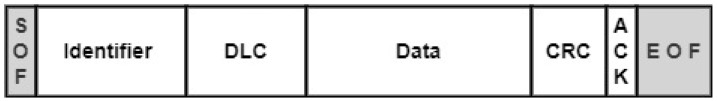
The standard structure of a CAN data frame.

As shown in Figure 2, the standard structure of a CAN data frame comprises several fields, and each field serves a specific purpose [23]:

- Start of Frame (SOF): a single dominant bit that marks the start of the CAN data frame.
- Identifier (ID): an 11- or 29-bit identifier that uniquely identifies the content and purpose of a message. It tells the receiving nodes what kind of data the message contains and can indicate the source or destination of the message.
- Data Length Code (DLC): a four-bit field that specifies the length of the data that are transmitted in bytes.
- Data: an array of up to eight bytes of actual data is transmitted. It is the main payload of the frame. For example, a vehicle may transmit information about engine speed, temperature, or brake status.
- Cyclic Redundancy Check (CRC): a 15-bit field used for error detection.
- Acknowledge (ACK): a bit that is transmitted by the receiver to acknowledge the receipt of a valid frame.
- End of Frame (EOF): a sequence of bits that marks the end of the CAN data frame.

It is important to understand that the specific interpretation of CAN frames, including their associated ECUs, as well as the information within the data field, is typically confidential and proprietary to vehicle manufacturers. This level of confidentiality is crucial due to the significant roles that these details play in the sophisticated functionality of a vehicle’s systems and features. Consequently, to effectively understand CAN bus traffic, one requires access to the CAN database (DBC) file specific to that vehicle’s manufacturer. This file contains the necessary definitions for decoding raw CAN data [26].

### 2.3. Vulnerabilities of Controller Area Network Protocol

The CAN protocol was originally designed for traditional isolated vehicles without considering security measures. As vehicles become increasingly connected, these vulnerabilities become potential targets that can be exploited by adversaries [24,27]. Firstly, its broadcast nature enables the easy interception of messages by adversaries. Secondly, the absence of authentication means that the sources of the messages cannot be verified, leaving the system vulnerable to malicious frames. Thirdly, the lack of encryption, where messages are transmitted in plain text, exposes message content to potential analysis and modification. Lastly, its identifier-based priority mechanism can be exploited for denial-of-service attacks; the identifier not only uniquely identifies a message but also determines its priority, with a smaller identifier signifying a higher priority. Thus, a malicious ECU can continuously transmit frames with the smallest identifier to prevent any legitimate ECU from transmitting valid frames. These vulnerabilities collectively compromise the security of the CAN protocol and facilitate various attacks, with the adversarial attacks discussed in this paper being of particular concern.

### 2.4. Adversarial Machine Learning

Adversarial machine learning (AML) is a research domain focused on developing robust ML models that can resist adversarial manipulation [3,28]. This domain covers the study of various adversarial attacks and defensive mechanisms relevant to deploying ML models in real-world scenarios [29].

Adversarial attacks employ advanced manipulation techniques to alter the decision-making process of ML models. These manipulations introduce subtle, typically undetectable changes to the input data of ML models, exploiting vulnerabilities within these models. The manipulated inputs are then called adversarial examples. According to Huang et al. [3], such attacks can be classified based on the stage of the attack: poisoning (or causative) attacks introduce manipulated inputs into the training data, corrupting the model’s learning process, while evasion (or exploratory) attacks involve manipulating inputs during the testing or inference phase, where the model is actively used, to mislead the model’s decision making. For instance, adversaries can deceive face recognition systems into misidentifying or failing to detect an individual by slightly altering facial features in an image. Additionally, the knowledge an adversary has about the ML system determines the type of attack [3]: white-box (WB) attacks, where the adversary has full knowledge of the system; gray-box (GB) attacks, with partial system knowledge; and black-box (BB) attacks, where the adversary does not know the system.

## 3. Related Work

Sun et al. [30] conducted a comprehensive survey discussing cybersecurity threats against CAVs, reviewing existing cybersecurity risks and vulnerabilities in this domain. They classified attacks against CAVs into several categories, one of which targets ML systems, highlighting the vulnerabilities that can be exploited. Our work lies at the junction of adversarial attacks and CAVs. In the sections that follow, we review the relevant literature in this area and position our work relative to the existing literature.

Adversarial attacks targeting ML models deployed in CAVs generally adopt one of two approaches, each distinguished by its medium of experimentation: digital or physical attacks. In digital attacks, the entire process—from the generation of adversarial examples to their introduction to the ML models—is conducted within the digital domain. In contrast, physical attacks directly interfere with the real-world environment. For instance, an adversary might place an adversarial sticker on a stop sign, causing an autonomous vehicle to misidentify it.

### 3.1. Digital Adversarial Attacks in CAV

Aung et al. [5] highlighted the problem of adversarial examples in autonomous vehicles. They showed that a convolutional neural network (CNN)-based traffic sign detection method could be evaded using adversarial methods such as the Fast Gradient Sign Method (FGSM) and Jacobian Saliency Method (JSMA). The German Traffic Sign Recognition Benchmark (GTSRB) dataset [31] was used to generate digital adversarial traffic signs, resulting in a reduction in the classifier accuracy from 98.77% to 55.88%. Despite the high success rate of the attack, the visual differences between the original and adversarial signs are noticeable. This could be due to the lack of restriction on the perturbation range while generating adversarial samples.

Patel et al. [6] demonstrated that despite a well-trained deep neural network (DNN) being robust to sensor noise, adversarial perturbations in sensor data can result in incorrect responses. Their experiment demonstrated that slight alterations in some Light Detection and Ranging (LiDAR) sensor data could lead to incorrect output from the DNN. The authors generated these perturbations on a LiDAR range image using various adversarial methods such as FGSM, iterative-FGSM, and iterative selective pixel modification (ISPM).

Sobh et al. [7] developed adversarial samples that target DNNs that perform multitask visual perception, that is, networks that perform multiple tasks, such as distance and motion estimation, by sharing specific layers and parameters. The experiments considered WB and BB settings using the FGSM and the evolution strategy algorithm (ES), respectively.

Choi and Tian [8] approached adversarial attacks against autonomous vehicles from a different angle. Unlike the work mentioned above, which employed classification loss to generate adversarial examples, Choi and Tian [8] explored the use of objectness loss—the likelihood of a bounding box containing an object—to create more effective adversarial examples. By applying the FGSM and projected gradient descent (PGD) techniques within the YOLO v4 detector [32], they demonstrated that adversarial examples crafted using objectness loss are more successful in disrupting object detection in autonomous vehicles than those based on other types of loss.

Xiong et al. [9] extended the concept of single-source adversarial attacks by introducing a multi-source attack strategy. The authors exploited the interdependence between image and LiDAR data to deceive the object detection models in autonomous vehicles. The experiments demonstrated that the proposed attack effectively compromises the perception models of these vehicles. While the attack produces robust adversarial examples, implementing it in real-world scenarios could present challenges.

### 3.2. Physical Adversarial Attacks in CAV

Kurakin et al. [10] were the first to transfer adversarial examples to the real world. Their experiment employed several adversarial methods, including the FGSM, Iterative Basic Method (IBM), and the Least-Likely Class (l.l.C) method, to generate adversarial images. These adversarial images were then printed, photographed using a cell phone camera, and fed into a classifier. The primary target model for their experiment was the ImageNet Inception classifier, which is based on CNN architecture. Their results demonstrated that these adversarial examples can deceive the CNN classifier even when captured through a camera in real-world conditions, not just in digital environments.

Sitawarin et al. [11] introduced two adversarial attacks targeting autonomous vehicle sign recognition systems. They assessed the effectiveness of these attacks in both digital and real-world contexts. The first attack, known as the out-of-distribution (OD) attack, enables adversaries to create adversarial examples from any location within the image space, that is, from any point outside the training or testing distribution. The second attack, the lenticular printing (LP) attack, exploits an optical phenomenon to fool the CNN-based sign recognition system.

Chen et al. [12] improved on earlier physical attacks on image classifiers by also including advanced object detection models. They introduced ShapeShifter, a new attack that creates physical changes to trick image-based object detectors like Faster R-CNN. ShapeShifter was able to generate adversarial stop signs that Faster R-CNN misclassifies as other objects, creating a risk for autonomous vehicles.

Similarly, Song et al. [13], whose work was further enhanced in [14], introduced the Robust Physical Perturbation (RP2) algorithm. This algorithm creates adversarial objects that object detection models either ignore or misclassify. By applying an adversarial poster over a stop sign, they ensured it went undetected by the models. In experiments, both the YOLO v2 detector [33] and Faster R-CNN were unable to recognize the modified stop signs.

Feng et al. [34] developed the MagMonitor system, a cost-effective, wireless, and environmentally friendly solution for on-road traffic surveillance using a single magnetic sensor. This system uses a magnetic model that varies with vehicle types to estimate speeds and classify vehicles by analyzing the magnetic waveforms from moving vehicles. The effectiveness of the MagMonitor system was confirmed through field experiments on real roads. While the system is effective in real-time traffic monitoring, the robustness of the magnetic model against attacks has not been explored. Consequently, adversarial attacks could be designed to manipulate the magnetic signatures of vehicles, potentially causing the MagMonitor system to misclassify them or inaccurately estimate their speeds.

### 3.3. Comparison with Existing Literature on CAVs

Undoubtedly, the issue of adversarial attacks against connected and autonomous vehicles remains a significant area of focus in both research and practical applications, as highlighted in recent studies [15,16]. Despite the progress made in understanding these attacks, existing research has focused predominantly on manipulating perception models in CAVs, driven by the significant success of adversarial attacks in the image domain. Perception and control systems (PCSs) enable vehicles to understand the environment by analyzing data from sensors like cameras and LiDAR, facilitating autonomous navigation. However, they do not explore other potential models within CAVs where adversarial attacks could be equally impactful, such as those related to cybersecurity tasks. Therefore, our work aimed to bridge this gap and expand the threat landscape of adversarial manipulation in CAVs. Specifically, we investigated the susceptibility of IDS models deployed within CAVs to such attacks, thus providing a more comprehensive perspective on the adversarial attacks faced by CAVs. This focus is crucial; as discussed in Section 1, the IDS’s placement within a critical location, namely, the IVN, highlights the severe implications of its compromise through adversarial attacks. Table 1 provides a summary of related work.

Adversarial attacks against an IDS differ from those on perception models in three key aspects. Firstly, due to the distinct functionalities of vehicles’ perception models versus IDS models, they process different types of data. Perception models generally handle image or LiDAR data, while IDS models analyze network frames. Manipulating network frames poses a greater challenge than images due to their stringent structural requirements; any manipulation could compromise their functionality. Secondly, the types of ML algorithms used vary: perception models generally use image-based algorithms, typically CNNs, whereas IDS models often utilize DNNs or their advanced versions. Thirdly, while adversarial attacks on perception models exploit the vehicle’s autonomous functions by modifying the environment (even though some experiments in previous research have been conducted digitally, due to the complexities involved in real-world execution), attacks against an IDS can be executed remotely, leveraging the vehicle’s connectivity features. By exploiting vulnerabilities in the vehicle’s external connections, adversaries can remotely inject adversarial frames into the IVN, potentially triggering erroneous IDS responses. This approach might be preferred by adversaries, as it bypasses the complexities associated with changing the physical environment perceived by the PCS.

Therefore, we believe that these distinctions underscore the need for rigorous research in this area and, thus, inevitably raise the following questions:

- RQ1: How vulnerable are IDSs trained on IVN data to adversarial manipulation, given the less complex and more predictable nature of IVNs as opposed to traditional and IoT networks, and considering that the efficacy of adversarial attacks typically relies on exploiting data dimensionality?
- RQ2: What adversarial methods enable the creation of a strong adversarial attack, specifically one that can deceive an IDS model in an IVN under black-box conditions, where the adversary has no prior knowledge of the model?
- RQ3: How do the inherent constraints of the IVN affect the process of crafting adversarial attacks in real-world scenarios?

## 4. Redefining Adversarial Perturbations for IVN-Based IDS

IDSs are intricately designed, reflecting the unique characteristics of the environments they protect. Each environment posits its own set of demands, resulting in various complexities in the implementation of IDSs. Understanding the fundamental differences between environments is crucial when considering their vulnerability to adversarial manipulations.

In this context, the IVN setting presents unique characteristics that distinctly define its susceptibility to such manipulations. These inherent characteristics can significantly influence the success or failure of adversarial attacks, particularly when compared with other network environments.

Firstly, analyzing structural differences reveals that the CAN frame is distinguished by its inherent simplicity, with a data length of up to 8 bytes per message. This design is optimized for short, real-time communications, meeting the needs of vehicular internal networks. Consequently, IDSs designed for this environment are tailored to focus on a limited set of features within these simple frames. In contrast, networks operating on the TCP/IP protocol, whether in traditional or IoT environments, require a more complex packet structure to support a broader range of communications.Therefore, an IDS for these networks must accommodate a wider array of features. For example, state-of-the-art IDS datasets, such as CICIDS [39], include more than 80 features, far exceeding the 10 features typically extracted from the CAN frame.

Secondly, considering the resource constraints of vehicles, IDSs deployed within the IVN are typically designed to ensure optimal performance while remaining lightweight. Conversely, IDSs for TCP/IP networks benefit from more computational resources, which leads to a more complex design.

Finally, in terms of built-in security, the CAN protocol inherently lacks comprehensive security features, making it particularly vulnerable to attacks if not adequately protected, as discussed in Section 2.3. In contrast, the TCP/IP protocol incorporates security protocols, such as TLS/SSL, providing significant benefits including confidentiality, integrity, and authentication to ensure secure communication.

The characteristics mentioned above have several implications for how an adversary can manipulate the CAN frame. Initially, the inherent simplicity of the CAN frame makes it a challenge to manipulate it in a way that can bypass an IDS under realistic constraints. Adversarial manipulation is proportional to space dimensionality, which refers to the number of features used to describe data points. In their foundational work, Goodfellow et al. [40] argued that adversarial examples often arise from linear behavior in high-dimensional spaces. More recently, Han et al. [41] reviewed theoretical explanations for these phenomena, noting that adversarial attacks are frequently facilitated by high data dimensionality. This is why the phenomenon has been extensively studied within the image domain, which is inherently high-dimensional.

Given the simple structure of the CAN frame, only a limited number of features are subject to manipulation. Protocol-related features cannot be manipulated without resulting in a malformed frame. Even if such a frame were to bypass the IDS, it would fail to function as intended. Consequently, our research investigated whether the simplicity and lower dimensionality of CAN frames in an IVN might inherently protect against such attacks. This led to our central research question: How vulnerable are IDSs trained on IVN data to adversarial manipulation, given the less complex and more predictable nature of IVNs as opposed to traditional and IoT networks, and considering that the efficacy of adversarial attacks typically relies on exploiting data dimensionality? Addressing this question is crucial as it may contradict the prevailing assumption that high dimensionality increases vulnerability to successful adversarial attacks. This potential contradiction could pave the way for further exploration of adversarial robustness in lower-dimensional models, such as those based on tabular data.

Additionally, resource constraints on IDSs within IVNs introduce further challenges. Specifically, these constraints necessitate a lightweight design. However, such a design might exclude certain features and layers, thereby reducing the number of ‘dark spots’—areas that adversarial methods often exploit to remain undetected.

Moreover, with the protocol’s minimal security mechanisms, the simplicity of the CAN protocol [27] can facilitate unauthorized access to the vehicle’s internal network, including the IDSs deployed therein. This situation has two main implications for the adversarial manipulation of IDSs. First, the absence of robust security measures could allow adversaries to remotely penetrate the vehicle’s internal network, enabling them to inject adversarial frames and compromise the IDSs. Previous research has demonstrated the feasibility of remotely accessing IVNs in several modern vehicles, such as a Jeep Cherokee [18], Tesla [19], and BMW [20], particularly as these vehicles evolve toward a higher degree of connectivity and autonomy, such as having internet connections. Second, given the widespread availability of vehicles, adversaries could potentially access a portion of the dataset used for IDS training. This might be accomplished through connections to the vehicle’s OBD-II port or the unauthorized integration of components into the IVN. Such data could in some way mirror the normal behavior patterns used to train the IDSs. The extraction of this data is crucial for creating a substitute model that mimics the targeted IDS, thereby facilitating the generation of adversarial samples from these frames with the substitute model. Therefore, compared to networks where access to training data is challenging, this scenario could increase the chances of successful adversarial manipulations.

The distinctive features of the IVN—specifically, its simplicity, constrained resources, and lack of inherent security—significantly influence how an adversary might manipulate a deployed IDS. While the simplicity and resource constraints can pose challenges to such manipulation, the absence of built-in security may ease the execution of attacks. In the following sections, we will explore how adversarial attacks against an IVN can be conducted considering these factors.

## 5. Materials and Methods

This section details the methodologies used to generate adversarial attacks against our targeted IDSs, which were designed specifically for IVN security. It outlines the threat model, the experimental setup, and the adversarial methods applied.

### 5.1. Threat Model

We investigated a scenario where an adversary aims to induce misclassification in an ML/DL-based IDS within an IVN by creating adversarial examples. These examples were crafted through minor manipulations of the IVN data frames and were remotely injected into the victim’s IVN by exploiting vulnerabilities in the vehicle’s external connections. Such vulnerabilities are mentioned in Section 1. This scenario is particularly relevant for connected and autonomous vehicles that interact with the external world.

To better compare this scenario with those in future work, we redefined it according to the taxonomy presented by Aloraini et al. [42]. This taxonomy categorizes adversarial attack scenarios using distinct characteristics, simplifying their evaluation and comparison. It also introduces the concept of ‘adversarial power’ as defined by Apruzzese et al. [43], which assesses attack feasibility based on the adversary’s control over five elements: training data, feature set, detection model, feedback, and manipulation depth. While more control increases the likelihood of success, it can also render the scenario unrealistic and not applicable in real-world settings.Therefore, we assign the adversary the lowest power level, which implies that if a weak adversary can manipulate the IDS, a stronger one would undoubtedly succeed as well.

**Figure 3:**
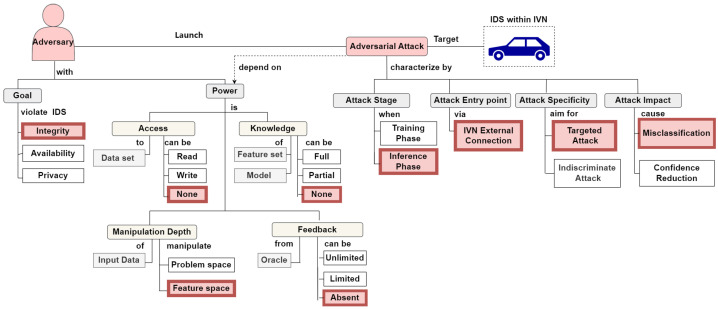
Threat model of the proposed adversarial attack against targeted IVN-based IDSs.

Figure 3 illustrates the adversary’s goal to compromise the integrity of the IDS by causing it to misclassify malicious frames as benign and vice versa. The adversary lacks access to the dataset used to train the IDS, gaining only limited insights from normal traffic data logged through the publicly accessible OBD-II port. These data, lacking in both volume and variety, especially in malicious samples, are not equivalent to those in training dataset. Additionally, the adversary is presumed to be unaware of the IDS’s feature set and configuration, treating the IDS as a ‘black box’. The adversary manipulates the CAN frame at the feature level while ensuring that the functionality of the CAN frame remains unaffected, categorizing this as a feature-space attack. IVN-based IDSs typically do not provide feedback to adversaries, such as labels, which constrains their ability to refine attacks, unlike in other applications.

Lastly, we assumed that the attack occurs during the inference phase after the IDS has been deployed, utilizing a vulnerable external IVN connection. The adversary’s objective is targeted misclassification: manipulating a frame so that the IDS erroneously assigns it to a specific class, potentially compromising vehicle safety.

It should be noted that Figure 3 presents a comprehensive taxonomy of adversarial attacks across various IoT applications [42]. Within this figure, we specifically highlight the settings of our proposed scenario—relevant to the IDS deployed in the IVN—in red to distinguish the scenario clearly.

### 5.2. Methodology

In line with our formulated threat model, we adapted the methodology initially proposed by Papernot et al. [44] to generate adversarial examples specifically designed for an IVN. This approach is based on the assumption that the adversary operates with zero knowledge about the model, essentially treating it as a black box.The adversary’s only ability is to observe the labels that the model assigns to selected inputs.

The fundamental concept is centered around the transferability phenomenon observed across different architectural model designs [4,40]. In other words, adversarial examples created using a substitute model often result in misclassifications not only from the substitute but also from the target model due to the similarities in their decision boundaries.

The methodology functions as follows: A substitute model, denoted as *S*, becomes instrumental in creating adversarial examples that *S* misclassifies. With complete knowledge of *S*’s parameters, the adversary can employ white-box adversarial attacks to generate samples that *S* misclassifies. In assuming that the transferability property holds between *S* and the target model *T*, examples designed for *S* are also likely to be misclassified by *T*.

In the IVN context, the adversary does not have direct communication with the IDS, hindering the ability to observe the IDS’s responses to selected inputs. However, a significant advantage in the IVN is the adversary’s capability to access some normal traffic through the OBD-II port. This access allows the adversary to build a dataset to train a substitute model and create adversarial examples, thus applying the methodology in a novel way. The adversary simply needs to add some malicious samples to the normal traffic collected through the OBD-II port to create a dataset suitable for training a substitute IDS.

### 5.3. Experimental Setup

All experiments were conducted using the Anaconda Jupyter Notebook environment, specifically Python version 3.9.16. To launch adversarial attacks, we utilized the implementation provided by the Adversarial Robustness Toolbox (ART) developed by IBM (Armonk, NY, USA) and the Linux Foundation AI [45]. ART, a Python library dedicated to machine learning security, offers resources for developers and researchers to assess and defend machine learning models against adversarial attacks. Among the available tools, ART offers the most comprehensive and up-to-date collection of adversarial attacks and defenses, which was the primary reason for our selection. The experiments were implemented on a desktop computer running Ubuntu, equipped with an Intel® Core™ i7-11700 (2.50 GHz × 16) processor, and 31 GiB of memory.

### 5.4. Dataset Description

The primary dataset used in this work was the car hacking dataset [38], which is extensively cited in the current literature [17]. It was provided by the Hacking and Countermeasure Research Lab (HCRL) and is publicly available for academic research. The dataset was carefully collected from a car during normal operation and under a series of attack experiments. It is divided into five distinct segments: one comprising normal driving data, and four others, each corresponding to a different form of an IVN attack, including DoS, fuzzing, and two types of spoofing attacks that affect the vehicle’s RPM and gear displays. For a detailed statistical breakdown of the dataset, see Table 2.

Data for normal driving conditions were collected to create a baseline of what standard car communication looks like, free of any attacks. The other subsets offer a combination of normal communication mixed with data under an attack injection. Each subset was collected over a timeframe lasting from 30 to 40 min [38]. The dataset has several attributes, including timestamps, CAN ID, DLC, actual payload [D0, D8], and a label that distinguishes between normal and malicious frames.

The selection of the dataset was driven by several factors. Primarily, the MTH-IDS [21], which served as one of our target models, was initially trained using this dataset. Additionally, the dataset includes a dedicated subset representing normal traffic, which resembles the data that an adversary could potentially collect through the OBD-II port, as discussed in Section 5.1.

### 5.5. Dataset Preprocessing

This section outlines the steps undertaken to prepare the dataset for subsequent use. The process involved the following several stages:

- Adjusting Label Misplacement: The original dataset comprised 12 columns: Timestamp, CAN ID, DLC, eight data field columns (D0 to D7), and a label column. A notable issue was the misplacement of labels when the DLC value was less than 8. Typically, frames with a DLC less than 8 would only partially fill the data fields (e.g., D0 to D3), leaving the rest null, which is expected since data field lengths vary from 0 to 8 bytes. However, we discovered that in such cases, labels were mistakenly inserted into the first null data field (e.g., D4) rather than in the designated label column. To address this, we developed an automated Python script to check the DLC of each row and reposition the labels correctly into the label column.
- Merging Subsets: The dataset was organized into distinct folders for each attack type: DoS, fuzzy, gear, and RPM spoofing, with each folder containing both normal and specific attack traffic. In this step, these subsets were merged into a comprehensive dataset that includes both normal traffic and four types of attack traffic. This integration facilitates the training of a single IDS capable of identifying all types of attacks, rather than separate IDSs for each attack category.
- Feature Selection: As previously discussed, the structure of the CAN data frame is relatively straightforward, offering limited options for feature selection. We utilized all features except the timestamp. This decision was based on the observation that timestamps were generated by the CAN logger and are not an intrinsic part of the frame. Consequently, timestamps have been generally disregarded in the literature unless the proposed detection mechanism explicitly requires their use.
- Hexadecimal to Decimal Conversion: The CAN ID and data fields (D0 to D7) were logged as hexadecimal values. To ensure compatibility with ML algorithms, these values were converted to decimal format.
- Label Encoding: The labels, which were categorical (normal, DoS, fuzzy, gear, and RPM), were encoded into numerical values using the LabelEncoder from Sklearn [46]. This step is essential because ML algorithms require numerical inputs.

These preprocessing steps are critical for the training of the baseline-IDS. It should be noted that the MTH-IDS [21] utilizes additional preprocessing steps, which will be detailed in a subsequent section.

### 5.6. Targeted IDS Training

Our experiment examined two distinct IDS models: a self-created baseline IDS and the MTH-IDS, as outlined in the recent literature [21]. We selected a baseline model to assess the vulnerability of IVN models to adversarial manipulations and to explore their feasibility within this domain. This was motivated by the observation that the success of adversarial attacks often correlates with the specific nature of the data.

To ensure our findings’ applicability across different model architectures, we tested generated adversarial examples on the MTH-IDS, deployed in IVN settings [21]. Our choice of the MTH-IDS was informed by its hybrid detection capabilities, combining signature- and anomaly-based methods to identify both known and unknown attacks, an area into which adversarial examples typically fall. Notably, the MTH-IDS is practical for real-time applications in in-vehicle networks: it processes network frames in just 0.509 ms, significantly under the 10 ms vehicular security threshold, and requires only 2.61 MB of memory, comfortably within the limits of standard vehicle-level machines with over 1 GB of RAM [21]. Furthermore, its comprehensive training encompasses the entire CAN data frame, enhancing its ability to detect a range of attacks, including those involving ID changes and payload manipulations [17]. The multi-tiered structure of MTH-IDS reflects the complexity of real-world models, requiring adversarial examples to overcome multiple tiers to cause damage.

#### 5.6.1. Baseline IDS Architecture

The baseline IDS employs a DL-based architecture, which includes an input layer, four hidden layers, and an output layer, as illustrated in Table 3. The choice of a DNN for the baseline IDS was driven by the aim to explore the feasibility of adversarial manipulation against both ML and DL models. This exploration is crucial as the MTH-IDS incorporates both supervised and unsupervised ML approaches. The baseline IDS was trained on a merged dataset consisting of four subsets, totaling 16,569,475 samples. This dataset included 14,237,958 normal and 2,331,517 malicious samples, specifically targeting DoS, fuzzy, gear, and RPM spoofing attacks.

Following the preprocessing steps outlined in Section 5.5, the dataset was split into a 70% training and a 30% testing division. The IDS classifies inputs into multiclass categories: normal, DoS, fuzzy, gear, or RPM spoofing. Given the dataset’s imbalance, the IDS’s performance was evaluated using the F1 score metric, with the results presented in Table 4.

#### 5.6.2. MTH-IDS Architecture

The MTH-IDS is a hybrid system that combines a signature-based IDS with an anomaly-based IDS, resulting in a multi-tiered architecture. Complementing the preprocessing steps outlined in Section 5.5, MTH-IDS incorporates an additional preprocessing layer as its first layer. This layer applies various customized processes, including data sampling, normalization, and feature engineering. The second layer, the signature-based component, utilizes four tree-based supervised learners—Decision Tree (DT), Random Forest (RF), Extra Trees (ET), and Extreme Gradient Boosting (XGBoost)—as multiclass classifiers to detect known attacks. This layer also includes a stacking ensemble model to enhance the performance of these supervised learners. The third layer employs cluster labeling (CL) with k-means as an unsupervised learner for zero-day attack detection. Additionally, this layer integrates two biased classifiers to minimize false negatives (FNs) and false positives (FPs). Similar to the baseline IDS, the MTH-IDS was trained on a dataset comprising 16,569,475 samples, including 14,237,958 normal and 2,331,517 malicious samples. For a detailed explanation of how the MTH-IDS was implemented, refer to [21]. Table 5 presents the detailed performance of MTH-IDS, where the F1 scores for the signature-based and anomaly layers are reported separately, as indicated in the original paper, to verify the accuracy of our re-implementation of the IDS. It is worth noting that the results presented for the signature-based layer were achieved using an ensemble stacking model, while the results for the anomaly-based layer were computed by averaging the outputs from the biased classifiers, similar to what was done in the original study.

**Figure 4:**
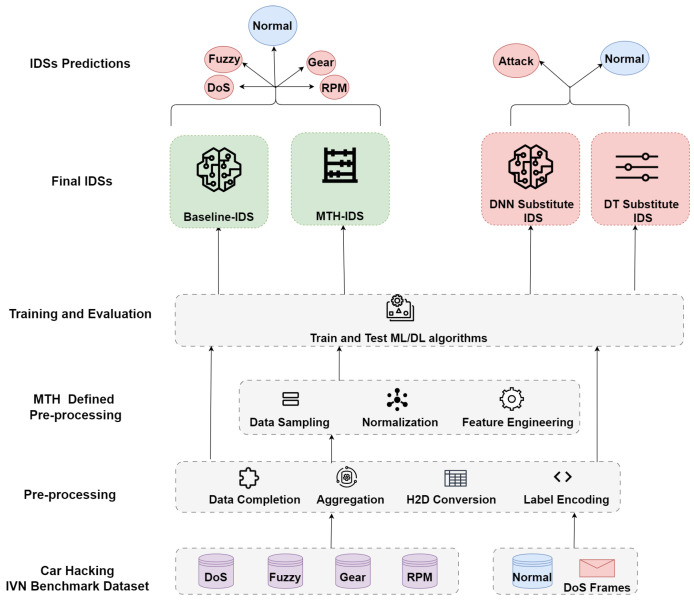
Step-by-step training and testing processes for targeted and substitute IDSs.

After achieving satisfactory performance metrics, we can now use the IDSs to evaluate the success of our adversarial examples. It is important to note that the training of these models involved the use of four distinct subsets, deliberately excluding the subset composed solely of normal data, which was reserved for subsequent adversarial manipulation. Figure 4 clearly outlines the steps taken to train the targeted IDS models.

### 5.7. Substitute IDS Training

Training a substitute IDS *S* to approximate the targeted IDS *T* presents significant challenges, primarily due to the need for acquiring extensive training data. These data are crucial for *S* to closely approximate the decision boundary of *T*.

We adhered to the scenario outlined in our threat model (see Section 5.1), where the adversary lacks access to the training dataset of *T*. The adversary typically gathers normal data either through the OBD-II port or unauthorized integration into the IVN, enabling them to monitor CAN traffic. To train *S*, it was necessary to integrate malicious samples into this dataset.

Our research utilized a previously collected dataset because acquiring real IVN data can be prohibitively expensive. This approach poses challenges in creating a dataset that closely mimics the original to train *S*. The primary dataset [38] included four types of attacks: DoS, fuzzy, gear, and RPM spoofing. Among these, only DoS attacks could be readily simulated and integrated into the substitute training dataset. This is feasible because an attacker, having analyzed the normal traffic, can identify the CAN IDs that, when targeted, could trigger a DoS attack. DoS attacks exploit the CAN protocol’s collision handling mechanism, where the frame with the smallest identifier gains control of the bus, allowing the adversary to easily augment DoS frames into the dataset.

Simulating other types of attacks without direct access to a real vehicle is nearly impossible, which, while a limitation, also enhances the realism of our substitute IDS. As *S* is trained on both normal and DoS data, and *T* handles multiple classes, this discrepancy could potentially hinder adversarial transferability. This configuration reflects real-world constraints, where *S* cannot perfectly replicate *T*, thereby deviating from typical WB settings.

To simulate these conditions, we used the normal data subset of our primary dataset, which was not included in training *T*, along with malicious samples exclusively from the DoS subset. Consequently, our dataset now comprised 988,871 normal samples and 587,521 DoS samples, totaling 1,576,392 samples. This extensive dataset supported the training of our substitute IDS *S*. Henceforth, we will refer to the dataset used to train *S* as the ‘substitute dataset’, distinguishing it from the original dataset used to train *T*.

#### 5.7.1. DNN Substitute IDS Architecture

The main substitute IDS employed a DNN architecture, consisting of an input layer, three hidden layers, and an output layer, as detailed in Table 6. The choice of a DNN for the substitute IDS was primarily driven by the requirements of most adversarial manipulation methods, which necessitate a differentiable model to generate adversarial samples. DNNs, being inherently differentiable, were thus preferred over traditional ML models for this task. Moreover, in considering the adversarial perspective, it was strategic to use a model that adversaries expect to mimic the IDS architectures deployed within vehicles. DNNs or their simpler variants are predominantly used in research that utilizes whole CAN frame features for detecting attacks [17]. The simplicity of DNNs, compared to more complex spatial and sequential models such as CNNs or recurrent neural networks (RNNs), makes them practical for use in scenarios with resource constraints, such as in vehicles.

The comprehensive nature of whole frame features allows for the detection of a wide range of attacks. Since our classification is based on whole CAN frame features, drawing comparisons with other DL techniques like CNNs, which are primarily utilized in perception models that interpret environmental cues from images, as discussed in Section 3, may not be justifiable. When applied to CAN frame data, CNNs require the transformation of CAN bus data into images, as observed in [47], or they focus solely on sequences identified with CAN IDs, as in the cases of [38,48,49]. For these reasons, our implementation exclusively employs a DNN model.

The DNN was trained on a substitute dataset to categorize the data as normal or indicative of DoS attacks. We refined the architecture of the DNN through multiple iterations, testing various parameters with the substitute dataset to ensure optimal performance.

#### 5.7.2. DT Substitute IDS Architecture

The second substitute IDS employs the DT algorithm. The development of this IDS was influenced by the specific requirements of the ART DecisionTree adversarial manipulation method, which exclusively accepts DT models for generating adversarial examples targeting tree-structured models. Additionally, considering that the targeted IDSs are presumed to be BB systems, an adversary may employ a variety of substitute IDSs to increase the likelihood of a successful attack. The DT model was trained using the DecisionTreeClassifier from Sklearn, with default parameters applied to the substitute dataset.

It is important to note that both substitute IDSs were trained on a substitute dataset, distinct from the primary dataset utilized for the targeted IDSs. Moreover, these substitute IDSs are binary classifiers, differing from the multi-class configuration of the targeted IDSs. Figure 4 illustrates the training steps for the substitute IDSs, while Table 7 summarizes their performance metrics.

### 5.8. Domain Constraints

As the targeted and substitute IDSs were implemented, the next phase involved the generation of adversarial examples. However, the initial step in this process required a comprehensive understanding of the domain constraints. The adversary’s objective extends beyond merely having the frame misclassified by the IDS; it also includes ensuring that the adversarial frame retains its functionality. Mbow et al. [15] emphasized that very few studies have accounted for these constraints when developing adversarial attacks on network traffic. They pointed out that due to these constraints, adversarial methods effective in other contexts may not perform as well in network environments, underscoring the need for further research to assess their viability in these settings.

In this work, we studied CAN traffic and identified three key constraints crucial for crafting adversarial examples in the context of IVNs. First, only a subset of CAN data frame fields can be modified. Second, amendments should be within each field’s permissible range and should not affect dependent fields, given the correlations between them. Lastly, adversarial perturbations must not violate the properties inherent to the original samples; that is, a malicious frame must retain its malicious intent.

The configurable features of the CAN data frame include the ID, DLC, and data fields. Although the CAN protocol transmits all data frames indiscriminately, regardless of their IDs, each ECU is programmed to respond exclusively to a predefined set of IDs relevant to its specific functions. Therefore, maintaining the ID feature in data frames is essential to ensure that adversarial frames are processed correctly by the ECUs. The DLC indicates the number of bytes in the data field and is closely correlated with it. According to the CAN protocol specification [23], only DLC values ranging from 1 to 8 are permissible; other values are not usable. Since the DLC reflects the number of bytes and is configured during the setup, it cannot be manipulated, assuming adversaries are manipulating existing frames that have already been logged from CAN traffic. Finally, the data field, consisting of 8 dynamic bytes, is the only part that can be realistically manipulated, providing adversaries with an opportunity for alteration.

To enforce these constraints, we use a boolean mask during the adversarial example generation process, where only data fields are marked “True” for manipulation, while other fields are not. Furthermore, we use clipping values to ensure that manipulations remain within the data field range of 0 to 255. After the generation process, compliance with these constraints is also confirmed through a subsequent post-generation check to ensure adherence. Table 8 summarizes the applied constraints.

### 5.9. Generating Adversarial Examples against Targeted IDSs

The adversarial manipulation problem is typically formulated as an optimization task aimed at identifying the minimal epsilon (i.e., the smallest perturbation) that causes the targeted IDS to misclassify an input into a targeted class. In this context, epsilon represents the magnitude of the perturbation applied to the input data.

Numerous methods have been identified in the literature to address this optimization task. Based on the literature reviewed in Section 3 and previous work [42] that explored various adversarial methods in IoT applications, this work included all relevant adversarial methods except those specifically designed for image, object, or voice data. The adversarial methods considered include FGSM [40], BIM [10], PGD [50], JSMA [51], DeepFool [52], C&W [53], EAD [54], and DecisionTree attack [55]. These methods vary in their methodologies for calculating the epsilon, which influences the speed and strength of the generated adversarial examples. For further details, refer to the respective papers.

The implementation of these adversarial methods using ART receives the victim IDS, original samples, and—for targeted attacks—a specified target label. The generation of adversarial examples begins with these original unaltered samples. The examples are refined iteratively, using the IDS at each stage to evaluate their effectiveness. This process ends when a sample is classified as the target label or reaches the limit of allowed iterations. In our BB setting, we provided these methods with substitute IDSs, the substitute dataset, and target labels that aimed to misclassify normal frames as attacks, and vice versa, alongside original labels for baseline comparisons. Once an example successfully deceives the substitute IDS, it is presented to the targeted IDSs. This leverages transferability, with the aim of inducing misclassifications in the targeted IDSs.

We applied the ART implementation to all adversarial methods uniformly, with the exception of the DecisionTree attack. This exception is due to the inherent design of the DecisionTree attack in ART, which does not support the application of a manipulation mask and allows the manipulation of all features. Specifically, the DecisionTree attack starts by identifying the path to the leaf node corresponding to the original class of the sample. It then searches for leaf nodes within the DT that have a different class label from the initial node. The path between these nodes is established, identifying the specific features that must be altered to induce a misclassification by the DT.

However, in constrained domains such as an IVN, not all features can be manipulated. Consequently, we introduced a manipulation mask to the perturbation process in the DT. This modification ensures that the adversarial path involves only features that can be manipulated, thus producing functional CAN frames. It is crucial to note that this constraint diminishes the method’s efficiency in generating adversarial examples, primarily because certain decision nodes critical to the path represent core features. If these core features are unmodifiable, the algorithm struggles to find a feasible adversarial path. Despite this reduction in efficiency, the importance of generating adversarial examples that are viable as functioning frames and not merely dismissed cannot be overstated. This adjustment is pertinent not only for IVN but also for other constrained domains.

While many parameters are common across different methods in ART, some parameters are unique to specific approaches.The epsilon value, denoted as ‘eps’, determines the maximum magnitude of each modification and is set within the range of 1 to 10, with the ‘eps_step’ fixed at 0.1. Although there is no standardized guideline in the literature for selecting ‘eps’, and its value is often unreported, it is essential for minimizing perturbations. Larger values of ‘eps’ may result in the generation of entirely new samples that are easily misclassified by the IDS. Consequently, ‘eps’ is constrained within the range of 1 to 10, incremented by 0.1 steps. The ‘clip_values’ parameter, defining the feasible range for each feature to ensure realistic modifications, and the ‘mask’ parameter, specifying which fields can be altered, are elaborated on in Table 8. The ‘norm’ parameter, determining the type of norm used to measure input data alterations, offers options such as infinity, 1, or 2. We conducted experiments using these three norms alongside each ‘eps’ value. Setting ‘targeted’ to true aims for a specific misclassification by the IDS, as discussed in Section 5.1.

The ‘theta’ and ‘gamma’ parameters in the JSMA control the amount of perturbation and the maximum fraction of features to perturb, respectively. The gamma value in ART, ranging from 0 to 1, governs the proportion of features perturbed, with 0 indicating no perturbation and 1 allowing all features to be modified. Intermediate values such as 0.3 permit the alteration of up to 30% of the features. Accordingly, ‘theta’ is set within the epsilon range from 1 to 10, while ‘gamma’ spans from 0 to 1. The ‘offset’ parameter in DecisionTree attacks serves a similar purpose to the ‘eps’ and ‘theta’ parameters and thus shares the same range as them.

**Figure 5:**
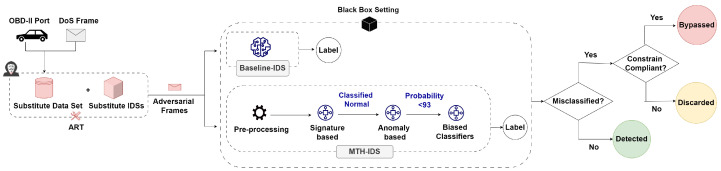
Pipeline of proposed adversarial attack against targeted IDS.

A sample is considered adversarial if it successfully evades the targeted IDSs while adhering to the practical constraints we discussed. Any sample that fails to meet these constraints is disregarded. As illustrated in Figure 5, the process of introducing adversarial examples into the targeted IDSs differs between the MTH-IDS and baseline IDS. While feeding adversarial examples to the baseline IDS is a more straightforward process due to its single-layer structure, in the MTH-IDS, adversarial examples undergo an initial preprocessing stage. Subsequently, they must satisfy specific conditions at each subsequent layer of the MTH-IDS before a final decision is made. This aspect will be discussed further in Section 6.

## 6. Results

In this section, we present our findings on the performance of targeted IDSs under adversarial settings, with a particular focus on two critical assumptions: the manipulation of malicious frames to be classified as normal and normal frames as malicious. The findings provide vital insights into the vulnerabilities of these IDSs, highlighting key considerations for designing robust IDS solutions for IVN environments.

To accurately evaluate the targeted IDSs, we initially provided a clean version of the substitute dataset to the IDSs and recorded their performances. This step enabled us to distinguish between samples misclassified by the IDSs under normal conditions (i.e., without any adversarial manipulation) and those that were true adversarial examples. After recording the performances with the clean dataset, we then introduced adversarial examples for further analysis.

The process of introducing adversarial examples into the targeted IDSs varied depending on the IDS, as shown in Figure 5. For the baseline IDS, adversarial examples were fed directly. In contrast, the MTH-IDS involved a multi-step procedure that included normalization and feature selection, where only four features were chosen by the MTH-IDS for sample classification. The process then moves to a signature-based layer. If a sample is classified as normal at this stage, it is deemed suspicious and forwarded to the next layer, which is the CL k-mean anomaly detection. If the clustering probability here is less than 93%, then the sample is passed to biased classifiers for the final decision [21].

### 6.1. Case 1: Malicious to Normal

In normal settings, as detailed in Table 9, the targeted IDSs successfully detected all malicious samples in the substitute dataset, recording zero false negatives (FNs). However, under an adversarial attack scenario, the baseline IDS showed significant vulnerability, failing to detect all adversarial DoS samples. In contrast, the MTH-IDS showed robustness, accurately identifying all manipulated DoS samples. The underlying reason for this includes the following. First, the substitute model, a DNN-based IDS, shares a more similar architecture and decision boundary with the baseline IDS, enhancing the transferability of adversarial examples. Second, unlike the baseline IDS, the MTH-IDS was trained to recognize unknown attacks, which contributes to its increased robustness against modified DoS attacks. Third, the signature-based layer of the MTH-IDS employs tree-based learners, and our analysis reveals that the CAN ID is the primary decision node for identifying a DoS attack. Preserving this ID, while complying with IVN constraints, results in samples being classified as DoSs, leaving no path for adversarial manipulation. This highlights the effectiveness of tree-based learners in detecting DoS attacks within IVNs. However, it is important to note that if an adversary selects a different small identifier, such as CAN ID = 2, for a DoS attack, the samples would bypass the signature-based layer of the MTH-IDS. Our tests using the ART version of the decisionTree attack (without modification constraints) confirmed this, showing that altering the CAN ID to 2 allows samples to evade the signature-based layer. However, due to the presence of an additional anomaly detection layer in the MTH-IDS, these samples were eventually detected. This situation emphasizes that, while tree-based learners are effective against DoS attacks, it is crucial to consider all potential identifiers that could facilitate such attacks.

### 6.2. Case 2: Normal to Malicious

In normal settings, as detailed in Table 9, the targeted IDSs demonstrate satisfactory performances, although with some false positives (FPs). The F1 scores were 95% and 88% for the baseline IDS and MTH-IDS, respectively. This is in part due to the inherent challenge of IVN-based systems in identifying fuzzy attacks, which are quite similar in nature to normal traffic. Additionally, the substitute dataset was considered new to the targeted IDSs, which means that we expected a lower performance.

Under an adversarial attack, the MTH-IDS performs worse, misclassifying 517,567 normal frames as malicious out of 988,871, with 226,443 already misclassified under normal settings. This accounts for approximately 52.34% of the total normal samples. Similarly, the baseline-IDS, while more robust, was also manipulated to misclassify 160,060 of 988,871, knowing that 145,186 were already misclassified under normal conditions. This represents approximately 16.19% of the total normal samples. This type of manipulation poses a greater danger, as the adversary does not need to possess any specific attack payload but only needs to ensure that any normal packet is manipulated to appear malicious. This can trigger an IDS response without any actual attack payload, leading to potentially higher risks depending on the manipulation frame’s priority.

In general, adversarial attacks against both targeted IDSs were successful in reducing the baseline-IDS’s F1 score from 95% to 38% and the MTH-IDS F1 score from 97% to 79%. This reduction was observed despite the proprietary nature of the IVN data, different decision boundaries between substitute and target IDSs, the encompassing binary and multiclassification, and varying model architectures.

Table 9 presents the performance metrics, specifically emphasizing the total number of samples that were misclassified by the target IDSs. For a detailed analysis of the misclassified samples in each layer of the MTH-IDS, refer to Table 10. It is important to note that the F1 scores reported in Table 9 represent the average scores of the classification layers based on signatures, anomalies, and biased classifiers. This explains why, at times, a lower F1 score is reported despite fewer misclassified examples. This occurs because if the biased classifiers perform poorly, it lowers the average score, even with a smaller number of misclassified samples.

## 7. Discussion

**Figure 6:**
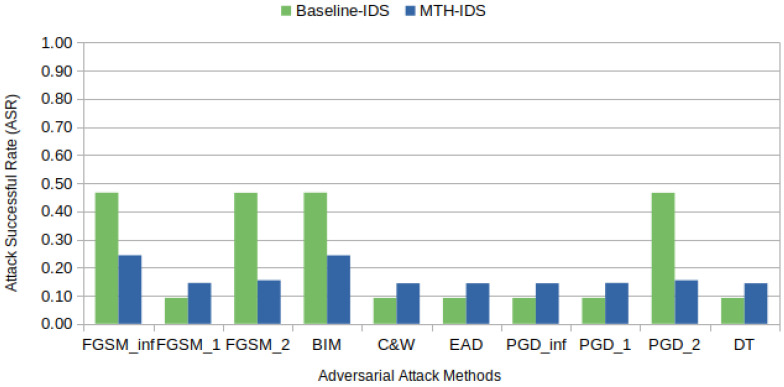
Attack success rate of adversarial methods applied to targeted IDSs with epsilon = 1.

**Figure 7:**
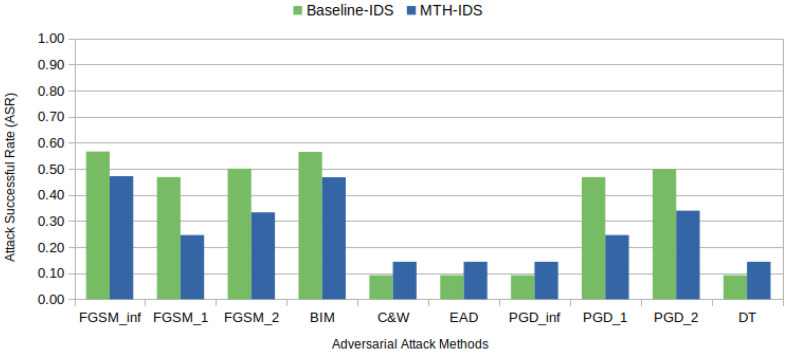
Attack success rate of adversarial methods applied to targeted IDSs with epsilon = 10.

FGSM and BIM have emerged as the most effective adversarial attack methods in terms of their ability to bypass targeted IDSs, as depicted in Figure 6 and Figure 7. These figures detail the attack success rates (ASRs) for various adversarial methods under two distinct levels of epsilon perturbations. The ASR [56], which measures the effectiveness of adversarial attacks, represents the ratio of successful adversarial samples to total attack attempts (1,576,392 in our case), with higher ASRs indicating more successful attack methods.

Figure 6 highlights FGSM and BIM as the most effective methods under an epsilon value of 1, achieving the highest ASR at approximately 47%. In contrast, C&W, EAD, and DT perform poorly, demonstrating their ineffectiveness at this epsilon level as they only misclassify samples that are already misclassified under normal conditions. Notably, PGD, in its various forms (PGD_inf, PGD_1, and PGD_2), exhibits moderate success rates that are superior to those of C&W and EAD but still significantly lower than those of FGSM and BIM. This suggests that while PGD shows some capability under low epsilon values, with norm-2 constraints, it does not perform as well as the leading method.

Figure 7 shows that when epsilon is increased to 10, there is a general improvement in the ASR for almost all methods as a result of the expanded perturbation magnitude. FGSM and BIM display the most substantial improvements, with the ASR nearing 60%. The PGD variants also show enhanced performances; however, they do not match the effectiveness of FGSM and BIM. This pattern indicates that while PGD methods benefit from an increased epsilon, their effectiveness in bypassing IDSs is less pronounced compared to FGSM and BIM. C&W, EAD, and DT remain less effective with no significant change. The detailed comparisons in Figure 6 and Figure 7 underscore the impact of epsilon scaling on the effectiveness of various adversarial attacks, suggesting that epsilon settings need to be carefully considered during adversarial robustness assessments as different levels may reveal more potent vulnerabilities.

C&W and its variant EAD perform poorly because they are designed to generate highly subtle adversarial examples with minimal perturbations. Considering the IVN constraints that further limit perturbations, and the fact that IDSs are typically adept at detecting minor deviations, these methods proved ineffective. In contrast, FGSM and BIM’s success in deceiving IDSs can probably be attributed to their ability to introduce more noticeable changes through a gradual and iterative process, bypassing the detection thresholds. Additionally, the IVN constraints significantly hinder the DecisionTree attack. Due to the inherent simplicity of CAN bus frames, the DecisionTree attack is restricted to a limited range of adversarial pathways. These constraints do not leave viable adversarial paths to be exploited.

This finding is significant because methods that introduce more noticeable changes, such as FGSM, BIM, and PGD, remain capable of generating adversarial samples that can deceive the MTH-IDS. To illustrate, during its preprocessing stage, the MTH-IDS selects only four features for decision making. This selection includes the CAN ID, which remains unchanged due to IVN constraints, unlike the baseline IDS, which considers all features in its decision process. Therefore, even though not all features are selected—a scenario expected in real-world applications due to the resource constraints of vehicles—these samples are still able to fool the IDS. Table 11 details the features manipulated by various adversarial methods to create examples aimed at deceiving the targeted IDSs. From Table 11, we observe that methods like C&W, EAD, and DT do not modify the original samples, highlighting their difficulty in finding an adversarial path under IVN constraints compared to the other methods.

JSMA and DeepFool were excluded from our primary analysis due to their inability to adhere to specific constraints. JSMA does not allow individual feature-clipping ranges, as it was originally designed for image data where all pixels share the same range. Similarly, DeepFool lacks control over the manipulation of specific features. Although these methods reported promising results, they were not included in our overall result calculations.

Lastly, while decision tree-based learners demonstrate greater robustness against an adversarial FN, they are vulnerable to an adversarial FP. As illustrated in Table 10, the signature-based and biased classifier layers in the MTH-IDS, responsible for all FPs, employ tree-based learners. In contrast, the DNN was more effective in managing adversarial FPs, despite having an architecture similar to those of the substitute models. This similarity indicates a substantial likelihood of adversarial sample transferability.

### 7.1. Answering Research Questions

RQ1: How vulnerable are IDSs trained on IVN data to adversarial manipulation, given the less complex and more predictable nature of IVNs as opposed to traditional and IoT networks, and considering that the efficacy of adversarial attacks typically relies on exploiting data dimensionality? When assessing the vulnerability of an IDS within an IVN, our research employed a methodology that involved both single-layer and multi-layer IDSs. These IDSs were rigorously trained on a range of known and unknown attacks. Contrary to expectations that the relative simplicity of IVNs might confer a degree of inherent security, our results reveal a significant susceptibility to adversarial manipulation even under a black-box setting with no knowledge about the IDS. Interestingly, our findings seem contrary to the common assertion, as noted in [40,41], in that the high dimensionality of data typically enhances vulnerability to adversarial attacks.

The findings provide essential insights into the vulnerabilities of ML/DL-based IDSs deployed in IVNs to adversarial manipulation, indicating a critical need for enhanced security measures within the CAV domain. It is especially significant that a recent survey [17] revealed that the prevalent literature often overlooks the robustness of IDSs within CAVs against adversarial attacks. Our results suggest that while IDS solutions may demonstrate high detection rates, they could paradoxically become the weakest link in IVNs if their susceptibility to adversarial attacks is not addressed. The implications of these vulnerabilities are profound—not only do they highlight the immediate need for deploying IDSs with considerations for adversarial robustness, but they also underscore the potential impacts on vehicle safety.

By incorporating adversarial robustness assessments of ML/DL IDSs within the constraints of the IVN environment, future research can build on these findings to develop more robust security solutions. This exploration contributes to the growing body of knowledge on adversarial strategies and offers a foundational approach for evaluating the robustness of IDSs within IVNs under black-box scenarios.

Furthermore, our findings provide a foundation for designing response systems in critical-safety environments, such as vehicles, where responding to attacks is as vital as their detection [57]. Therefore, developing IDS response mechanisms that consider and counteract potential adversarial manipulations is essential for protecting passengers and preventing malicious exploits that could undermine vehicle integrity and safety. Such manipulations could trigger actions harmful to both the vehicle and its occupants. Addressing these concerns mitigates the risk of false positives and promotes a safer transition toward fully autonomous and connected transportation systems.

RQ2: What adversarial methods enable the creation of a strong adversarial attack, specifically one that can deceive an IDS model in an IVN under black-box conditions where the adversary has no prior knowledge of the model? The results indicate that both the FGSM and its iterative version, BIM, when applied through the substitute model methodology, were notably effective in deceiving the targeted IDSs. This outcome suggests that even with limited insight into the targeted IDS models, these methods can compromise the integrity of an IDS in IVNs. Refer to Section 7 for an explanation of why these methods performed better than the others.

RQ3: How do the inherent constraints of IVNs affect the process of crafting adversarial attacks in real-world scenarios? The nature of an IVN, discussed in Section 4, limits the success of adversarial attacks in two key ways. First, the simplicity of network traffic poses a challenge for specific adversarial methods. This is evident in the DecisionTree attack, where such simplicity results in no viable adversarial paths. Additionally, constraints on the range and features that must remain unchanged reduce the effectiveness of more sophisticated adversarial methods, such as JSMA, DeepFool, C&W, and EAD, which have proven powerful in other domains like image manipulation. These methods typically require a degree of flexibility and feature manipulation that IVNs inherently restrict, thereby reducing their applicability and effectiveness in this context. Secondly, the resource constraints of IVNs pose another challenge for adversaries. For instance, in the MTH-IDS, only four features are selected among all the CAN data frame features during the preprocessing step. This means that even if an adversary attempts to manipulate certain features, these may not be selected, and hence, the adversarial manipulation fails to deceive the targeted IDSs.

### 7.2. Limitations

In the literature, IVN-based IDSs have been developed using features that include CAN ID, CAN Payload, the combination of CAN ID and Payload (CAN frame), and physical characteristics such as vehicle voltage signals [17]. In our work, we focused solely on IDSs based on the CAN frame. This approach not only aids in detecting changes in IDs and manipulations in payload but also, according to a recent survey by Rajapaksha et al. [17], is the most extensively studied in the literature, having the largest number of publications compared to other types of IDSs.

Although the proposed method has demonstrated success in deceiving targeted IDSs, it has certain limitations. Our focus was primarily on the manipulation of DoS attacks, specifically within scenarios of malicious-to-normal misclassification. However, this approach was not extended to other types of attacks, such as fuzzy and spoofing attacks.

This limitation arises because the adversary is assumed to log CAN traffic either from the OBD-II or from unauthorized devices connected to the IVN. Under these conditions, the adversary can understand network operations, determine the IDs and their priorities, and thus predict the IDs used for DoS attacks, allowing them to effectively manipulate the DoS frames. However, for fuzzy and spoofing attacks, the adversary must first generate and inject these attacks into the IVN, observe the outcomes, and then manipulate these frames before feeding them to the targeted IDSs. Since we used a public dataset that was already collected, only the manipulation of DoS frames was feasible. Additionally, we could not utilize the existing fuzzy and spoofing samples in the dataset due to the black-box scenario constraints, which presume that the adversary has no knowledge of the training dataset.

These limitations lead us toward potential avenues for future improvement in adversarial manipulation methods within IVNs. Future research could explore the susceptibility of other types of IDSs deployed in IVNs to adversarial manipulations. Additionally, extending our work to include not just the manipulation of DoS attacks but also fuzzy and spoofing attacks could provide a more comprehensive understanding of IDS vulnerabilities. This could involve developing methods to simulate and manipulate these attack types under white-box, grey-box, and black-box scenarios using substitute models, which would enhance our methods for evasion in adversarial attacks.

Another important direction could involve the evaluation of scenarios involving poisoning adversarial attacks in the context of CAVs. Research could focus on how such attacks might affect learning processes and decision making within IDSs, potentially leading to the development of more robust learning algorithms that are less susceptible to adversarial manipulations.

Moreover, developing response systems that account for adversarial manipulations presents a critical area for future research. Such systems would require the integration of real-time detection and response strategies that not only identify but also react appropriately to adversarial manipulations, thereby ensuring vehicle safety even when under attack. This integration of responsive measures is crucial for maintaining the integrity and safety of vehicles against evolving adversarial threats.

## 8. Conclusions

The rapid advances in CAVs are driven by significant developments in ML and DL. However, the inherent susceptibility of these technologies to adversarial attacks poses substantial risks. While existing research predominantly focuses on manipulating perception models in CAVs, particularly in the image domain, our research shifts the focus toward the cybersecurity aspects. We examined the vulnerability of ML/DL-based IDSs in IVNs to adversarial manipulations, aiming to stay one step ahead of adversaries.

In this study, we implemented two representative IDS models: a baseline IDS and a state-of-the-art IDS, MTH-IDS, which served as our targets. Operating under black-box conditions, with no prior knowledge of the target IDSs, we employed a substitute IDS and data from the publicly accessible OBD-II port to initiate adversarial attacks, focusing on generating FNs and FPs in traffic detection. Our proposed adversarial method significantly reduced the F1 score of the baseline IDS from 95% to 38% and of the MTH-IDS from 97% to 79%. Scenarios that generated FPs proved particularly effective, revealing that even IDSs trained to detect unknown attacks, such as the MTH-IDS, often overlook adversarial FP samples. This oversight poses a significant risk, especially given the accessibility of the OBD-II port.

Looking ahead, our findings lay the groundwork for two pivotal future directions: enhancing the robustness of IDSs in IVNs against adversarial attacks and developing novel response strategies in CAVs that specifically address adversarial attacks leading to FPs. As CAVs are increasingly integrated into our transportation systems, ensuring their cybersecurity against adversarial manipulations is not merely a technological challenge, it is a fundamental requirement for their safe and reliable operation.

## Figures and Tables

**Table 1:** Adversarial attacks on CAVs: summary of the related work.

Ref.	Threat Model	Adversarial Method	Target	ML/DL Model	Dataset	Data Type
[5]	GB	FGSM and JSMA	PCS	CNN	GTSRB [31]	Image
[6]	WB	FGSM and ISPM	PCS	DNN	Proprietary Dataset	Range Image
[7]	WB and BB	FGSM and ES	PCS	DNN	Woodscape [35]	Image
[8]	WB	FGSM and PGD	PCS	YOLO v4 [32]	KITTI [36] and COCO [37]	Image
[9]	WB	GAN ^1^ and VAE ^2^	PCS	——	KITTI [36]	Images and LiDAR Data
[10]	WB	FGSM, BIM, l.l. Class	PCS	CNN	ImageNet	Image
[11]	WB and BB	OD and LP	PCS	CNN	GTSRB [31]	Image
[12]	WB	ShapeShifter	PCS	Faster R-CNN	COCO [37]	Image
[14]	WB and BB	RP2	PCS	Faster R-CNN and YOLO v2 [33]	Proprietary Dataset	Image
Our Work	BB	Substitute Model	IVN	DNN and MTH-IDS [21]	Car Hacking [38]	Network Frame

^1^ Generative adversarial network. ^2^ Variational autoencoder.

**Table 2:** Statistical breakdown of the car hacking dataset [38].

Attack Type	Normal	Malicious	Total
DoS	3,078,250	587,521	3,665,771
Fuzzy	3,347,013	491,847	3,838,860
Gear Spoofing	3,845,890	597,252	4,443,142
RPM Spoofing	3,966,805	654,897	4,621,702
Normal	988,871	0	988,871
Total	15,226,829	2,331,517	17,558,346

**Table 3:** Baseline—IDS parameters.

Parameter	Value
Number of Neurons in Input Layer	10
Number of Hidden Layers	4
Number of Neurons in Hidden Layer	16
Number of Neurons in Output Layer	5
Epoch	10
Activation Function Hidden Layer	relu
Activation Function Output Layer	softmax
Optimizer	adam
Batch Size	32
Loss Function	categorical_crossentropy

**Table 4:** Baseline —IDS performance metrics.

Attack Type	Precision	Recall	F1 Score
Normal	99%	100%	99%
DoS	100%	100%	100%
Fuzzy	99%	99%	99%
Gear Spoofing	100%	100%	100%
RPM Spoofing	99%	100%	100%
Weighted Average	99%	99%	99%

**Table 5:** MTH-IDS performance metrics.

MTH-IDS Layer	Attack Type	F1 Score
Signature-Based	DoS	99%
	Fuzzy	99%
	Gear Spoofing	99%
	RPM Spoofing	99%
Weighted average		99%
Anomaly-Based	DoS	100%
	Fuzzy	78%
	Gear Spoofing	100%
	RPM Spoofing	99%
Weighted average		94%
MTH-IDS Average		97%

**Table 6:** DNN substitute IDS parameters.

Parameter	Value
Number of Neurons in Input Layer	10
Number of Hidden Layer	3
Number of Neurons in Hidden Layer	16
Number of Neurons in Output Layer	2
Epoch	50
Activation Function Hidden Layer	relu
Activation Function Output Layer	softmax
Optimizer	adam
Batch Size	10
Loss Function	categorical_crossentropy

**Table 7:** Substitutes IDSs’ performance metrics.

Substitute Model	Precision	Recall	F1 Score
DNN-based IDS	100%	100%	100%
DT-based IDS	100%	100%	100%

**Table 8:** Constraints applied in the generation of adversarial examples for car hacking dataset [38].

CAN Frame Field	Range	Modification	Mask
CAN ID	[0, 1068]	No	False
DLC	[1, 8]	No	False
D0	[0, 255]	Yes	True
D1	[0, 255]	Yes	True
D2	[0, 255]	Yes	True
D3	[0, 255]	Yes	True
D4	[0, 255]	Yes	True
D5	[0, 255]	Yes	True
D6	[0, 255]	Yes	True
D7	[0, 255]	Yes	True

**Table 9:** Comparative analysis of targeted IDSs’ performance under benign and adversarial attacks, highlighting normal-to-malicious and malicious-to-normal detection.

Evaluation Samples Distribution		Adversarial Dimensions				Benign Setting			Adversarial Setting		
Normal Sample	Malicious Sample	Adv. Method	Substitute Model	Target Model	Epsilon	Malicious to Normal (FN)	Normal to Malicious (FP)	F1 Score	Malicious to Normal (FN)	Normal to Malicious (FP)	F1 Score
988,871	587,521	FGSM (inf)	DNN	Baseline-IDS	1	0	145,186	95%	587,521	147,180	44%
				MTH-IDS		0	226,443	88%	0	383,797	81%
				Baseline-IDS	10	0	145,186	95%	587,521	305,246	38%
				MTH-IDS		0	226,443	88%	0	744,010	79%
		FGSM (1)	DNN	Baseline-IDS	1	0	145,186	95%	0	145,570	95%
				MTH-IDS		0	226,443	88%	0	228,598	86%
				Baseline-IDS	10	0	145,186	95%	587,521	150,655	44%
				MTH-IDS		0	226,443	88%	0	387,975	77%
		FGSM (2)	DNN	Baseline-IDS	1	0	145,186	95%	587,521	146,565	44%
				MTH-IDS		0	226,443	88%	0	244,132	96%
				Baseline-IDS	10	0	145,186	95%	587,521	202,113	42%
				MTH-IDS		0	226,443	88%	0	524,913	72%
		BIM	DNN	Baseline-IDS	1	0	145,186	95%	587,521	147,193	44%
				MTH-IDS		0	226,443	88%	0	383,635	81%
				Baseline-IDS	10	0	145,186	95%	587,521	303,465	38%
				MTH-IDS		0	226,443	88%	0	737,271	77%
		PGD (inf), C&W, EAD	DNN	Baseline-IDS	1	0	145,186	95%	0	145,186	95%
				MTH-IDS		0	226,443	88%	0	226,443	88%
				Baseline-IDS	10	0	145,186	95%	0	145,186	95%
				MTH-IDS		0	226,443	88%	0	226,443	88%
		PGD (1)	DNN	Baseline-IDS	1	0	145,186	95%	0	145,571	95%
				MTH-IDS		0	226,443	88%	0	228,588	86%
				Baseline-IDS	10	0	145,186	95%	587,521	150,682	44%
				MTH-IDS		0	226,443	88%	0	387,881	76%
		PGD (2)	DNN	Baseline-IDS	1	0	145,186	95%	587,521	146,563	44%
				MTH-IDS		0	226,443	88%	0	244,045	78%
				Baseline-IDS	10	0	145,186	95%	587,521	200,442	42%
				MTH-IDS		0	226,443	88%	0	535,280	72%
		DT	DT	Baseline-IDS	1	0	145,186	95%	0	145,186	95%
				MTH-IDS		0	226,443	88%	0	226,443	88%
				Baseline-IDS	10	0	145,186	95%	0	145,186	95%
				MTH-IDS		0	226,443	88%	0	226,443	88%

**Table 10:** Breakdown of adversarial example detection across MTH-IDS layers.

MTH-IDS Layer	Adv. Method	Epsilon	Misclassified Samples under Normal Settings	Misclassified Samples under Adversarial Settings	Successful Adversarial Examples	Successful Adversarial Samples Category	Suspicious Samples Passed to Next Layer
Signature-Based Layer	FGSM (inf)	10	225,877	743,922	518,045	Normal to Malicious (FP)	244,949
CL k-mean Layer			0	0	0	None	214
Biased Classifier Layer			566	88	88	Normal to Malicious (FP)	
Signature-Based Layer	FGSM (1)	10	225,877	387,700	161,823	Normal to Malicious (FP)	601,171
CL k-mean Layer			0	0	0	None	389
Biased Classifier Layer			566	275	275	Normal to Malicious (FP)	
Signature-Based Layer	FGSM (2)	10	225,877	524,652	298,775	Normal to Malicious (FP)	464,219
CL k-mean Layer			0	0	0	None	347
Biased Classifier Layer			566	261	261	Normal to Malicious (FP)	
Signature-Based Layer	BIM	10	225,877	737,181	511,941	Normal to Malicious (FP)	251,690
CL k-mean Layer			0	0	0	None	212
Biased Classifier Layer			566	90	90	Normal to Malicious (FP)	
Signature-Based Layer	PGD (inf) C&W EAD DT	10	225,877	225,877	0	None	None
CL k-mean Layer			0	0	0	None	None
Biased Classifier Layer			566	566	0	None	
Signature-Based Layer	PGD (1)	10	225,877	387,598	161,721	Normal to Malicious (FP)	601,273
CL k-mean Layer			0	0	0	None	396
Biased Classifier Layer			566	283	283	Normal to Malicious (FP)	
Signature-Based Layer	PGD (2)	10	225,877	535,021	309,144	Normal to Malicious (FP)	453,850
CL k-mean Layer			0	0	0	None	343
Biased Classifier Layer			566	259	259	Normal to Malicious (FP)	

**Table 11:** Features manipulated under various adversarial methods.

Attack	ID	DLC	D0	D1	D2	D3	D4	D5	D6	D7
FGSM	x	x	✓	✓	✓	✓	✓	✓	✓	✓
BIM	x	x	✓	✓	✓	✓	✓	✓	✓	✓
C&W	x	x	x	x	x	x	x	x	x	x
EAD	x	x	x	x	x	x	x	x	x	x
PGD	x	x	✓	✓	✓	✓	✓	✓	✓	✓
DT	x	x	x	x	x	x	x	x	x	x

## Data Availability

The data set presented in this study is publicly available in the Hacking and Countermeasure Research Lab repository at: https://ocslab.hksecurity.net/Datasets/CAN-intrusion-dataset (accessed on 12 May 2024) and has been cited in the manuscript.

## Abbreviations

The following abbreviations are used in this manuscript:

TLA	Three-letter acronym;
LD	Linear dichroism;
ML	Machine learning;
DL	Deep learning
IoT	Internet of Things;
IVN	In-vehicle network;
CAN	Controller area network;
ECU	Electronic control units;
OBD	On-board diagnostic;
WB	White box;
GB	Grey box;
BB	Black box;
ART	Adversarial Robustness Toolbox;
DNN	Deep neural network;
CNN	Convolutional neural network;
FGSM	Fast Gradient Sign Method;
BIM	Basic Iterative Method;
PGD	Projected Gradient Descent;
JSMA	Jacobian Saliency Map Attack;
C&W	Carlini and Wagner;
EAD	Elastic Net Attack;
TCP/IP	Transmission control protocol/internet protocol;
TLS	Transport layer security;
SSL	Secure socket layer;
RPM	Revolutions per minute;
FP	False positive;
FN	False negative;
ASR	Attack success rate.
